# Social–emotional functioning in young people with symptoms of eating disorders: A gender inclusive analogue study

**DOI:** 10.1002/brb3.2017

**Published:** 2021-01-09

**Authors:** Ashley Boscoe, Rebecca Stanbury, Amy Harrison

**Affiliations:** ^1^ Department of Psychology and Human Development Institute of Education University College London London UK

**Keywords:** anorexia nervosa, bulimia nervosa, eating disorders, emotion recognition, emotion regulation, emotional functioning, nonclinical, social functioning, young people

## Abstract

**Introduction:**

Contemporary models of eating disorders (EDs) suggest that EDs are maintained by social–emotional difficulties. However, supporting evidence is derived largely from female, clinic‐based samples. This study, which refrained from gender specific inclusion criteria, aimed to improve understanding of social–emotional functioning in a large community‐based analogue sample of young adults aged 16–26.

**Methods:**

Five hundred and forty‐four participants (85.1% female; mean age 21, *SD* = 4.3) completed the Eating Attitudes Test, Clinical Outcomes in Routine Evaluation, Difficulties in Emotion Regulation Scale, Social Phobia Inventory, Revised Social Anhedonia Scale, Toronto Alexithymia Scale, and the Reading the Mind in the Eyes Task.

**Results:**

One hundred and sixty‐four participants scored over the EAT‐26 clinical cutoff, and a two‐way multivariate analysis of covariance found a medium‐sized, statistically significant main effect of group on social–emotional functioning (*F*(5, 530) = 6.204, *p* ≤ .001, Wilks' Λ = 0.945, *d* = 0.48.), suggesting that individuals with significant ED symptoms found it more challenging to notice, label, and regulate emotions in themselves and recognize emotions in others. Gender did not significantly impact social–emotional functioning (*F*(10, 1,060) = 0.556, *p* = .850, Wilks' Λ = 0.990), and there was no significant group by gender interaction (*F*(10, 1,060) = 0.688, *p* = .737, Wilks' Λ = 0.987).

**Conclusion:**

These data suggest that the social–emotional difficulties, particularly with emotion recognition and regulation, present in clinical samples are also evident in young people of all genders with significant disordered eating. Future work could aim to recruit an even more gender‐diverse community sample to further elucidate social–emotional functioning in individuals in the community with significant disordered eating.

## Graphical abstract

This study aimed to improve understanding of social emotional functioning in a large community‐based analogue sample of young adults aged 16–26 with no gender specific inclusion criteria. Five hundred and forty‐four participants (85.1% female; mean age 21, *SD* = 4.3) completed the Eating Attitudes Test, Clinical Outcomes in Routine Evaluation, Difficulties in Emotion Regulation Scale, Social Phobia Inventory, Revised Social Anhedonia Scale, Toronto Alexithymia Scale, and the Reading the Mind in the Eyes Task. Data show a medium‐sized, statistically significant main effect of group on social emotional functioning, suggesting that individuals with significant ED symptoms find it more challenging to notice and label and regulate emotions in themselves and recognize emotions in others. Gender did not significantly impact social emotional functioning skills.

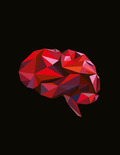

## 1 INTRODUCTION

The UK Royal College of General Practitioners (RCGP, 2013) has identified eating disorders (EDs) including anorexia nervosa (AN), bulimia nervosa (BN), binge eating disorder (BED), and other specified feeding or eating disorders (OSFED) as priorities for youth mental health. Approximately 13% of young people will experience an ED by the age of 20 (Culbert et al., 2015), and 15%–47% of young people will experience cognitions and behaviors associated with disordered eating, without meeting criteria for diagnosis or presenting in services (Culbert et al., 2009, 2015). Although current ED research has largely focused on females, (Strother et al., 2012), increasing numbers of males are reporting ED symptoms, with community studies suggesting males comprise approximately 25% of individuals who meet full diagnostic criteria (Sweeting et al., 2015). However, relatively few studies have included gender‐diverse samples, and thus, more work is needed to better understand how EDs might affect functioning across genders.

The social–emotional domain is an area of functioning which patients with EDs find challenging. This domain encompasses a broad range of skills, and one model proposed by Ochsner (2008) suggests that social–emotional functioning might involve the recognition of emotions in oneself and others and theory of mind, emotion regulation, and expression, the inference of emotional states from others’ bodily cues, social skills such as mimicry, and the acquisition of social‐affective values and responses (conditioning and reward learning). Patients with EDs report difficulties with emotion recognition (Oldershaw et al., 2011), emotion regulation (Monell et al., 2018), a reduced drive to seek out and experience pleasure from social interactions (social anhedonia; Harrison et al., 2014; Tchanturia et al., 2012), work and social functioning difficulties (Harrison et al., 2014; Patel et al., 2016), and small social networks (Westwood et al., 2016). Further discussed in contemporary models of EDs like the cognitive interpersonal maintenance model (Treasure & Schmidt, 2013), these inefficiencies in social–emotional functioning are also thought to maintain acute illness and have been found to lead to ED behaviors such as bingeing, purging, and restriction (Fairburn, 2008; Wonderlich et al., 2015). However, many previous studies have focused on collecting data from clinical samples (e.g., see reviews by Caglar‐Nazali et al., 2014; Dingemans et al., 2017; Oldershaw et al., 2011, 2015; Rienecke, 2018) and have somewhat neglected the large group of individuals in the community with significant ED symptoms. This makes it difficult to ascertain whether these social–emotional challenges are present only in clinical samples, or also affect the significant number of individuals with symptoms of EDs in the community not yet known to services. Collecting data from individuals in the community with significant symptoms is a form of analogue design which can help to better understand phenomena implicated in the development and maintenance of disorders like EDs. This design has previously been utilized by some researchers interested in social–emotional functioning in individuals in the community with ED symptoms. For example, in a small study from the UK, Jones et al. (2008) used scores from the Eating Attitudes Test (EAT‐26; Garner et al., 1982) to group female undergraduate students into high (*n = *29) or low (*n = *23) ED symptom groups. Emotion recognition was measured using the Facial Expression Recognition Task (FERT: Harmer et al., 2003) and those in the high symptom group were less accurate at recognizing happy and neutral faces than those with minimal symptoms. These findings are corroborated by another small study also from the UK in which Ridout et al. (2010) measured emotion recognition using the Awareness of Social Inference Test (McDonald et al., 2011) in females with high (*n* = 23) and low (*n = *22) scores on the Eating Disorder Inventory (EDI; Garner et al., 1983). Those who reported significant ED symptoms recognized significantly fewer emotional expressions than those with minimal symptoms. Another example from Goldschmidt et al. (2017) in 588 community‐based adolescent females found that emotion regulation difficulties contributed to losing control over eating. Unfortunately, the conclusions of these otherwise helpful studies on nonclinical populations are frequently limited to small samples of cisgender, heterosexual females.

Some studies that have included males have found that they may be protected from some of the social–emotional difficulties experienced by females with EDs. For example, Goddard et al. (2014) found that a clinical sample of 29 adult males with EDs from the UK did not differ from 42 males without EDs in their ability to recognize complex emotions and sensitivity to social threat, measured using the Reading the Mind in the Eyes (RME; Baron‐Cohen et al., 2001) and Emotional Stroop (Ashwin et al., 2006) tasks.

Some larger analogue studies have also included males and report different results. In a study of 296 undergraduate male students in the USA, Lavender and Anderson (2010) found that self‐reported difficulties in emotion regulation, particularly difficulties accepting emotional responses and using emotion regulation strategies, measured using the Difficulties in Emotion Regulation Scale (DERS; Gratz & Roemer, 2004) explained a small (1.3%) but significant proportion of variance in disordered eating, measured using the Eating Disorders Examination Questionnaire (EDE‐Q; Fairburn & Beglin, 1994). These data highlight that social–emotional functioning could be a challenge for males. A further study (Whiteside et al., 2007) from the USA which administered the DERS to 695 undergraduate psychology students, of which 41% (*n* = 284) were male, found that self‐reported difficulties in emotion regulation explained a greater proportion of variance in binge eating than gender, food restriction, and over‐evaluation of weight and shape, measured using the Eating Disorders Diagnostic Scale (EDDS; Stice et al., 2000). These findings suggest that social–emotional functioning might be a salient factor in males with ED symptoms. However, an additional issue with these data is that no analogue studies on social–emotional functioning have recruited individuals representing a broader range of gender identities or sexual orientations. The limited research that has been conducted in this population suggests there may be elevated ED risk in Lesbian Bisexual Gay Transgender Queer and gender nonconforming (LGBTQ+) individuals (Diemer et al., 2015; Feldman & Meyers, 2007; McClain & Peebles, 2016). This calls for ED research that includes gender‐diverse and sexual minority samples to further elucidate the extent of the association between LGBTQ + groups and ED pathology.

Therefore, this study aimed to recruit an inclusive analogue sample of young people of any gender identity, reporting a range of sexual orientations to understand whether individuals in the community with significant ED symptoms experience greater difficulties with social–emotional functioning than those without ED symptoms and whether difficulties in social–emotional functioning vary according to gender.

The first hypothesis was that there would be a significant main effect of group on social–emotional functioning (emotion recognition, measured using the RME Task Baron‐Cohen et al. (2001)), emotion regulation, measured using The DERS Short Form (DERS‐SF; Kaufman et al., 2016), alexithymia, measured using the Toronto Alexithymia Scale (TAS; Bagby et al., 1994); social anhedonia, measured using the Revised Social Anhedonia Scale (RSAS; Chapman et al., 1976), and social anxiety, measured using the Social Phobia Inventory (SPIN; Connor et al., 2000), such that those with ED symptoms will demonstrate greater difficulties in social–emotional functioning than non‐ED controls. We did not expect to find a main effect of gender on social–emotional functioning, nor a significant group by gender interaction effect on social–emotional functioning.

## 2 METHODS

### 2.1 Participants

Online and in‐person snowball (a nonprobability sampling technique where people who have previously participated help to recruit future participants through sharing information about the study either online or in person from among their acquaintances) and cluster sampling (a sampling technique in which naturally existing groups are sought out within a population; e.g., those with and without ED symptoms) techniques were used to obtain the sample over a 3‐month period through initially advertising on social media platforms, online forums, and charities. The inclusion criteria were not limited by gender, and participants were included if they had access to a computer with an internet connection, were aged 16–26 and able to read and respond to questions and tasks in English. When asking people to report on their gender identity and sexual orientation, these questions were set up as free text boxes so that participants did not have to find a category to conform to, but could instead report their gender identity in the way that made most sense to them. Overall, 624 participants responded to the advert and began the study; 80 (13%) were excluded as they did not meet inclusion criteria or did not complete at least 75% of the measures. The final sample consisted of 544 participants.

### 2.2 Measures

#### 2.2.1 Eating disorder symptoms

The Eating Attitudes Test, EAT‐26 (Garner et al., 1982), is a 26‐item screening tool that measures ED symptoms and, with an accuracy rate of at least 90%, can differentiate between those with and without EDs (Mintz & O'Halloran, 2000). The EAT‐26 provides three subscales; dieting, bulimia and food preoccupation and oral control and participants are asked to respond to items on a 6‐point scale, ranging from 0 (never) to 3 (always). Scores of ≥20 indicate high risk of an ED. When the measure is scored, responses need to be recoded for items 1–25 so that always receives a score of 3, usually receives a score of 2, often receives a score of 1 and sometimes, rarely and never receive a score of 0. For item 26, always, usually and often receive a score of 0, sometimes receives a score of 1, rarely receives a score of 2, and never receives a score of 3. Cronbach's alpha for this measure is 0.90 in those with AN (Garner et al., 1982). Cronbach's alpha for this sample was 0.92. Data on weight and height were requested to calculate body mass index (BMI; $\text{BMI}=\frac{\left(\text{weight}\;\text{i}\;\text{kilograms}\right)}{\text{heigh}\;\text{in}\;{\text{meters}}^{2}}$).

#### 2.2.2 Comorbidity

The Clinical Outcomes in Routine Evaluation (CORE‐10; Evans et al., 2000) is a 10‐item brief outcome‐screening tool assessing global mental health distress, including commonly experiences symptoms of anxiety and depression. Items are scored on a 5‐point scale from 0 (“not at all”) to 4 (“most or all of the time”), with scores >20 indicating “moderate‐to‐severe” distress. This measure has a Cronbach's alpha coefficient of 0.90 (Barkham et al., 2013). Cronbach's alpha for this sample was 0.84.

### 2.3 Social–emotional functioning measures

The DERS‐SF **(**Kaufman et al., 2016) is an 18‐item scale that assesses difficulties in emotion regulation across six subscales; Non‐Acceptance, Goals, Impulse, Awareness, Strategies, and Clarity. Participants respond on a 5‐point scale, ranging from 1 (almost never) to 5 (almost always). This measure has a Cronbach's alpha of 0.91 (Kaufman et al., 2016). Cronbach's alpha for this sample was 0.88. The total score was used as the outcome variable in this study.

The SPIN (Connor et al., 2000) is a 17‐item measure assessing social phobia across the spectrum of fear, avoidance, and physiological symptoms, rated on a scale from 0 (not at all) to 4 (extremely). Higher scores correspond to greater distress, and a score of >19 distinguishes between people with and without social phobia. This measure has a Cronbach's alpha of 0.94 (Connor et al., 2000). Cronbach's alpha for this sample was 0.93.

The RSAS (Chapman et al., 1976) is a 40‐item scale used to assess social anhedonia: diminished interest or pleasure in most or all social activities. Answers are indicated via a forced choice where participants are required to indicate if each statement is true or false for them. A “true” statement gives 1 point, while a “false” statement gives 0 points; a score of ≤12 indicates functionally impairing social anhedonia (Pelizza & Ferrari, 2009). This measure has a Cronbach's alpha of 0.95 (Fonseca‐Pedrero et al., 2009). Cronbach's alpha for this sample was 0.88.

The TAS‐20 (Bagby et al., 1994) is a 20‐item self‐report measure assessing the ability to label one's own emotions across 3 subscales (describing feelings; identifying feelings; and externally oriented thinking). Participants respond on a five‐point Likert scale ranging from 1 (strongly disagree) to 5 (strongly agree). Scores ≥ 61 indicate significant alexithymia, and scores between 52 and 60 indicate possible alexithymia. Scores ≤ 52 indicate the absence of alexithymia (Bagby et al., 1994). This measure has a Cronbach's Alpha of 0.86 (Parker et al., 2003). Cronbach's Alpha for this sample was 0.84.

The RME (Baron‐Cohen et al., 2001) is an experimental task of emotional recognition. Participants view 36 photographs of the eye area of the face and are asked to select from four options, the one that most closely matches what the person in the picture is thinking and feeling. The final score is the sum of the correct responses, and higher scores indicate better emotion regulation skills. Although this measure has been found to have poor internal consistency and convergent validity in its long form (Olderbak et al., 2015), it was selected due to its frequent use in the ED literature, because it could be administered online easily to the large sample we aimed to reach and because Olderbak et al., (2015, p17) recommend that a short form version with improved reliability and validity is suitable for measuring the construct in “unimpaired healthy adults” and this study involved recruiting people with ED symptoms who were expected to find this task more difficult than non‐ED controls.

### 2.4 Procedure

Participants learnt about the study through adverts posted on social media (Facebook, Twitter), web forums (Reddit, Craig's List), websites (Call for Participants, Gumtree), charity websites (Men Get Eating Disorders Too and Student Minds, Beat), and posters displayed in charities in London (Survey Circle and St. Christopher's Fellowship). We particularly focused on advertising on web forums where gender identity was being discussed to try to increase the range of genders we represented in our sample.

Participants completed the measures on the Qualtrics platform. They were provided with an information sheet and the research team's contact details to request further information if needed. Participants were then asked to provide written, informed consent. Participants were then asked to report on their age, gender, ethnicity, sexual orientation, nationality, and weight and height. They then completed the social–emotional self‐report measures and the Reading the Mind in the Eyes experimental task. Participants did not receive any financial reward or compensation for participation. The study received ethical approval from the University College London, Institute of Education Research Ethics Committee, reference 2316.23.

### 2.5 Data analysis

The independent variable of ED group was derived from the EAT‐26 data. Those in the ED group were individuals who reported ED symptoms on the EAT‐26 reflecting a score ≥20 on the EAT‐26; Garner et al., 1982. The non‐ED control group included those who scored below this cutoff (Non‐ED control group). Data were assessed for assumptions of normality using skewness and kurtosis values and histograms. Data violated normality assumptions for the factor level female on the z values for skewness (*z* > 2), but not for kurtosis (*z* = ±7). Three moderate outliers were identified initially using boxplots which on inspection represented valid responses and histograms showed an approximate normal distribution. Given these findings and the large sample size, parametric tests were selected. An independent samples *t* test was conducted to compare general mental health difficulties (CORE‐10) between the ED and non‐ED control groups. The ED group scored higher on this measure (*M* = 19.01, *SD* = 7.21) than the non‐ED control group (*M* = 12.59, *SD* = 6.57; t(542) = −10.164 , *p* ≤ .001). Therefore, to control for the potential confound of general mental health difficulties, the CORE‐10 was included as a covariate in subsequent analyses. A two‐way multivariate analysis of covariance (MANCOVA) was used to test the hypotheses. Group (ED/non‐ED controls) was entered as the independent variable. The TAS‐20, RME, DERS‐SF, SPIN, and RSAS were entered as dependent variables. The CORE‐10 score was entered as a covariate. Independent post hoc *t* tests were used to further explore main effects controlling for the CORE‐10. Missing data points were retained, and imputation was not used. Cohen's *D* was used as an estimation of effect size with 0.2 = small, 0.5 = medium, and 0.8 = large (Cohen, 1988). Data were analyzed using SPSS version 22.

## 3 RESULTS

The final sample consisted of 544 participants with a mean age of 21 (*SD* = 4.3; range 16–26 years). The mean BMI was 23.15 (*SD* = 7.2; range: 12.6–50.4).

Table 1 provides demographic data for the sample. A chi‐square test of independence showed that there was no significant association between ED risk group and gender *X* ^2^ (2, *N* = 544) = 5.05, *p* = .08. There was no significant association between ED risk group and nationality *X* ^2^ (10, *N* = 544) = 7.22, *p* = .70, ethnicity *X* ^2^ (4, *N* = 544) = 5.98, *p* = .20, nor sexual orientation *X* ^2^ (1, *N* = 544) = 3.27, *p* = .07. While not statistically significant, the estimated prevalence of young people at risk of developing an ED (EAT‐26 scores of ≥20) for the whole sample was 30.1% (*N* = 164). Of those at risk of developing an ED, 3.7% (*N* = 6) identified as “other,” 88.4% (*N* = 145) as females, and 7.9% (*N* = 13) as males. Within the ED risk group, 6.7% (*N* = 11) described their sexual orientation as “other,” 6.7% (*N* = 11) as homosexual, 20.1% (*N* = 33) as bi/pansexual, and 64.0% (*N* = 105) as heterosexual; 2.4% (*N* = 4) of participants did not disclose their sexual orientation.

**TABLE 1:** Demographic data for all participants

Gender			Ethnicity			Nationality			Sexuality		
*N* %			*N* %			*N* %			*N* %		
Female	463	85.1	Asian	58	10.7	American	241	43.3	Heterosexual	366	67.3
Male	67	12.3	Black	6	1.1	Asian	7	1.3	Homosexual	29	5.3
Other^a^	14	2.6	Caucasian	426	78.3	Australian	15	2.8	Bisexual/ Pansexual	116	21.3
			Mixed	41	7.5	British	147	27	Other^d^	25	4.6
			Other^b^	12	2.2	Canadian	37	6.8	Not reported	8	1.5
						Indian	2	0.4			
						Non‐British^c^ European	63	11.6			
						Other	7	1.3			
						South American	8	1.5			
						South East Asian	13	2.4			
						UAE	3	0.6			

^a^ Other gender identities include Nonbinary/Neutral, Gender Fluid, Agender, Transsexual, Transmasculine/Feminine, Questioning, and Unsure.

^b^ Other ethnicities include Indian, Afghani, Tamil Sri Lankan, Latina, Persian, and Mexican.

^c^ Other Non‐British European nationalities include Portuguese, German, Italian, Dutch, Finland, Sweden, and Turkish.

^d^ Other sexualities include Not sure, Fluid, Mostly straight, Homflexible, and Bicurious.

The MANCOVA showed a medium‐sized, significant main effect of group on social–emotional functioning, controlling for general mental health symptoms (*F*(5, 530) = 6.204, *p* = ≤0.001, Wilks' Λ = 0.945, *d* = 0.48.), suggesting that there were differences between the ED and non‐ED groups on the social–emotional functioning measures. There was no significant main effect of gender on emotional functioning (*F*(10, 1,060) = 0.556, *p* = .850, Wilks' Λ = 0.990), suggesting that social–emotional functioning skills did not vary between genders. There was no significant gender by group interaction effect (*F*(10, 1,060) = 0.688, *p* = .737, Wilks' Λ = 0.987), suggesting that social–emotional functioning did not vary as a function of both group status or gender.

Table 2 provides the means and standard deviations for the social–emotional functioning measures for the ED and non‐ED control groups.

**TABLE 2:** Descriptive statistics for ED risk condition and non‐ED control on the Toronto Alexithymia Scale, the Reading the Mind in the Eyes task, the Difficulties in Emotion Regulation Scale, Social Phobia Inventory, and Revised Social Anhedonia Scale

Measure	Whole sample *N* = 544			Eating disorder group *N* = 164 (EAT‐26 score ≥ 20)			Noneating disorder control group *N* = 380 (EAT‐26 score ≤ 19)		
	Mean (*SD*)	95% Confidence interval for mean		Mean (*SD*)	95% Confidence interval for mean		Mean (*SD*)	95% Confidence interval for mean	
		Lower	Upper		Lower	Upper		Lower	Upper
Alexithymia (TAS)	49.81 (13.56)	48.67	50.96	55.51 (13.84)	53.38	57.65	47.33 (12.67)	46.05	48.62
Emotion recognition (RME)	76.77 (11.22)	75.83	77.77	70.07 (10.15)	68.51	71.64	79.68 (10.39)	78.63	80.74*
Emotion regulation (DERS‐SF)	50.33 (10.07)	49.48	51.18	56.12 (10.075)	54.56	57.67	47.81 (8.99)	46.90	48.72**
Social anxiety (SPIN)	28.45 (15.18)	27.16	29.73	35.59 (15.49)	33.20	37.98	25.34 (13.96)	23.92	26.75
Social anhedonia (RSAS)	21.49 (3.62)	21.19	21.80	22.31 (3.74)	21.74	22.89	21.14 (3.52)	20.78	21.49

Abbreviations: DERS‐SF, Difficulties in Emotion Regulation Scale (short form); RME, Reading the Mind in the Eyes task; RSAS, Revised Social Anhedonia Scale; *SD*, standard deviation; SPIN, Social Phobia Inventory; TAS, Toronto Alexithymia Scale.

* Significant difference between the ED and non‐ED group *p* < .05.

** Significant difference between the ED and non‐ED group *p* < .001.

As shown in Table 2, controlling for general mental health symptoms (CORE‐10), those in the ED group reported significantly higher difficulties in emotion regulation than non‐ED controls (DERS‐SF; *F*(1, 541) = 15.25, *p* = ≤0.001, *d* = 0.33), greater difficulties in recognizing emotions in others than non‐ED controls (RME; *F*(1, 538) = 78.57, *p* = ≤0.001, *d* = 0.76), and there was a small‐sized increase in social phobia in those with EDs relative to non‐ED controls (*F*(1, 541) = 6.23, *p* = .013, *d* = 0.21). The groups did not differ regarding self‐reported alexithymia (TAS; *F*(1, 541) = 2.70, *p* = .101, *d* = 0.014) or social anhedonia (RSAS; *F*(1, 541) = 0.604, *p* = .437, *d* = 0.06).

## 4 DISCUSSION

This study aimed to investigate whether an analogue sample of young people, inclusive of all gender identities, with ED symptoms experience greater difficulties with social–emotional functioning than a non‐ED control group.

### 4.1 Social–emotional functioning

The first hypothesis, which was that there would be a significant main effect of group on social–emotional functioning, was partially supported by the data. In line with previous literature, those with ED symptoms had greater social–emotional difficulties than the non‐ED control group, particularly in relation to emotion regulation, measured using the DERS‐SF and recognizing emotions in others, measured using the RME, with small to medium effect sizes (Garner et al., 1982; Oldershaw et al., 2011). However, the groups did not differ significantly on the ability to recognize and label their own emotions (measured using the TAS), social anxiety (measured using the SPIN), or the desire to seek out and experience pleasure from social interactions (social anhedonia, measured using the RSAS). Our findings suggest that those at risk of developing ED’s report difficulties in emotional functioning, in line with previous findings by Oldershaw et al., (2011) and Monell et al., (2018). The current study did not find any statistically significant difficulties in social functioning to support previous literature that highlights social anhedonia and reduced social networks in clinical samples of females with EDs (Harrison et al., 2014). Therefore, this study is in partial concordance with the cognitive interpersonal maintenance model of EDs (Treasure & Schmidt, 2013), which suggests difficulties in social–emotional functioning maintain acute illness. It is possible that this may be due to the studies targeted sample of young people in the community who may not be in the acute stages of illness.

### 4.2 Gender and sexual orientation

As expected, and in keeping with the two previous large‐scale analogue studies which included males (Lavender & Anderson, 2010; Whiteside et al., 2007), all genders reported similar levels of social–emotional functioning difficulties and these difficulties affected people with ED symptoms similarly, regardless of their gender. These findings contradict Goddard et al.’s (2014) whose small clinical sample also undertook the RME task. It may be that there is greater variance in males in these skills, and larger samples are needed to identify these differences.

While there were no statistically significant differences in ED risk according to gender, the prevalence estimates for this sample suggest a higher number of females 88.4% (*N* = 145) were at risk of an ED than those who identified as male 7.9% (*N* = 13) and “other” 3.7% (*N* = 6). Our study showed a lower prevalence of males with EDs compared with a previous community sample in which estimates were approximately 25% (Sweeting et al., 2015), this may be due to the comparatively small number of males (*N* = 70) who took part in the current study.

Although there was no statistically significant association between ED risk group and sexual orientation, prevalence estimates for this sample suggest that some groups within the LGBTQ + population may be at elevated risk of ED pathology. This is in line with previous findings (Diemer et al., 2015; Feldman & Meyers, 2007; McClain & Peebles, 2016). For example, 20.1% (*N* = 33) of those who scored above the clinical cutoff on the EAT‐26 identified as bi/pansexual, 6.7% (*N* = 11) identified as homosexual, and 6.7% (*N* = 11) reported their gender identity as Nonbinary/Neutral, Gender Fluid, Agender, Transsexual, Transmasculine/Feminine, Questioning, and Unsure. With limited data around ED prevalence within the LGBTQ + community (Feldman & Meyers, 2007), it is difficult to make meaningful comparisons. However these data may indicate elevated risk of ED development within this population, particularly within bi/pansexual individuals. Further research is needed to corroborate these claims with robust statistical analysis and to better elucidate the determinates of ED pathology within this specific population.

### 4.3 Clinical implications

The findings highlight the need for clinicians to carefully consider ED risk and social–emotional functioning in people of all gender identities, not just cis females. One area in which this could be particularly useful is primary care, for example, by improving awareness of ED risk in males and other gender identities for General Practitioners and clinicians within the UK’s Improving Access to Psychological Therapies services. The hope is that this would lead to earlier identification of ED pathology and improved pathways to specialist treatment for people of all genders.

Furthermore, targeting emotion recognition and regulation may be an important means of preventing the development of more insidious forms of ED. Existing emotion regulation (ER) interventions have been identified as a useful transdiagnostic treatment for young people with EDs (Sloan et al., 2017, 2018); and our results support the need for further piloting of ER interventions for young people in the broader community.

In particular, support services that have regular contact with young people in the community, such as Teachers and Educational Mental Health practitioners (EHMP’s) in schools and practitioners based in University wellbeing services, may be well positioned to implement ER interventions within educational settings as a means of ED prevention.

Further, these findings suggest that clinicians working with people of all gender identities should consider their social–emotional functioning within specialist ED treatment.

### 4.4 Strengths and limitations

The study was successful to some degree in its aim of recruiting a significant group of males with ED symptoms. However, despite our best efforts during the recruitment phase, only 2.6% (*n* = 14) of the sample identified as noncisgender, disclosing their gender identity as nonbinary/neutral, gender fluid, agender, transexual, transmasculine/feminine, questioning, and unsure. Nevertheless, we were somewhat more successful in representing a range of different sexual identities in our sample and were able to include 170 individuals reporting their sexual identity as homosexual, bisexual, pansexual, or other, which fulfilled an important aim of the study.

The study is limited by its cross‐sectional design and reliance on largely self‐report measures. Further work might want to follow‐up the sample to explore social–emotional functioning in those with experience of EDs from a longitudinal perspective to better understand how the social–emotional difficulties might contribute to the onset of EDs. It would also be of value to corroborate the presence of ED symptoms using a clinical interview and to include a wider range of experimental measures of social–emotional functioning alongside the RME task to corroborate the self‐report data. While we put significant effort into reaching out to a gender‐diverse cohort, our sample of noncisgender individuals was relatively low. However, we hope that we have been successful in highlighting the need to include a broader range of gender identities in ED research, particularly as we found the social–emotional difficulties affected all genders equally in this study.

## 5 CONCLUSION

Our results suggest that young people in the community of all gender identities with significant disordered eating report higher levels of emotion regulation difficulties and find it more difficult than their unaffected to peers to recognize emotions in others. These social–emotional factors might contribute to the onset of EDs requiring clinical intervention, and future studies using longitudinal designs are needed to further explore these findings.

## CONFLICT OF INTEREST

The authors have no conflicts of interest to declare.

## AUTHOR CONTRIBUTIONS

Ashley Boscoe (AB), Rebecca Stanbury (RS), and Amy Harrison (AH) designed the study, AB and RS collected the data, AB analyzed the data, and AB and AH wrote the manuscript. All authors approved the submission.

### Peer Review

The peer review history for this article is available at https://publons.com/publon/10.1002/brb3.2017.

## Data Availability

The data that support the findings of this study are available from the corresponding author upon reasonable request. Please contact a.harrison@ucl.ac.uk for a copy of the anonymized data and a data dictionary.
